# Erratum: Von-Wolff et al. Testing of Eluates from Construction Products for Potential Environmental Effects Due to the Behaviour of *Enchytraeus albidus*. *Materials* 2021, *14*, 294

**DOI:** 10.3390/ma14092446

**Published:** 2021-05-08

**Authors:** Marya Anne von Wolff, Dietmar Stephan

**Affiliations:** Group of Building Materials and Construction Chemistry, Department of Civil Engineering, Technische Universität Berlin, Gustav-Meyer-Allee 25, 13B, 13555 Berlin, Germany; vonwolff@tu-berlin.de

In the original version of our article [1], the author Stephan Pflugmacher was included as an author, described as having made contributions in conceptualization, data curation, review and editing. The author Stephan Pflugmacher did not wish to be listed as co-author as his contribution was too small and therefore; we here correct the authors list and contributions, withdrawing the name of the author Stephan Pflugmacher.

Corrected authors list of this publication: Marya Anne von Wolff and Dietmar Stephan.

The authors apologize for any inconvenience caused and state that the scientific conclusions are unaffected.

## Data Availability

Data availability within the article.

## References

[1] Von-Wolff M.A., Pflugmacher S., Stephan D. (2021). Testing of Eluates from Waterproof Building Materials for Potential Environmental Effects Due to the Behavior of *Enchytraeus albidus*. Materials.

